# Northern Ohio Dental Association

**Published:** 1881-09

**Authors:** 


### NORTHERN OHIO DENTAL ASSOCIATION.

The annual meeting of the Northern Ohio Dental Association, was held May 10th and 11th in the dental depot rooms of Mr. Cogswell, Cleveland.

At 10 o'clock, May 10th, the association was called to order by the president, Dr. Chas. Buffett, and after the reading of the minutes of the previous meeting, and the transaction of the regular business, the subjects selected were taken up and discussed in their order during the day and evening sessions.

It is gratifying to be able to report an increased interest, manifested by an increased attendance, and a general disposition on the part of the members to participate in the discussions.

The dentist who fails to avail himself of the practical information to be gained in the discussion of the various subjects coming up for consideration in these meetings, lacks the progressive spirit, which every intelligent patient has a right to demand at his hands.

We bespeak for these meetings a still greater degree of interest, and a cordial invitation is extended to all who can possibly avail themselves of this means of profit and social intercourse, which is calculated to promote a fellow feeling, and do away with the petty jealousies which are among the greatest obstacles to progress.

The following officers were elected for the ensuing year:

President, Dr. Chas. Buffett, Cleveland,
Vice President, E. E. Rogers, Hudson,
Recording Secretary, A. J. Douds, Canton,
Corresponding Secretary, H. F. Harvey, Cleveland,
Treasurer, J. E. Robinson, Cleveland,
Board of Examiners, Drs. Lyder, Jennings, and Bell.

The following members were chosen as delegates to the American Dental Association:

Drs. H. L. Ambler, H. F. Harvey, I. Brown, L. Buffett, J. W. Lyder, Corydon Palmer, E. J. Way, G. W. Wilson, A. J. Douds, and E. W. Poole.

Executive Committee:

Drs. Harvey, Ambler, and Robinson.

The committee appointed to select subjects for discussion, reported no subjects suggested, and it was decided to wait the usual time before preparing the programme, and any member wishing a subject inserted, if sent to the chairman, Dr. Harvey, Cleveland, O., before April, 1st., will be considered by the committee.

A vote of thanks to Dr. Ketchem for anatomical specimens. Also to Drs. Butler and Vorce, for exhibition of microscopical specimens.

A vote of thanks was tendered Mr. Cogswell for use of rooms.

On motion, adjourned to next annual meeting to be held in Cleveland, O.

